# Necrosis related HIF-1α expression predicts prognosis in patients with endometrioid endometrial carcinoma

**DOI:** 10.1186/1471-2407-10-307

**Published:** 2010-06-19

**Authors:** Laura MS Seeber, Nicole Horrée, Petra van der Groep, Elsken van der Wall, René HM Verheijen, Paul J van Diest

**Affiliations:** 1 Department of Gynaecological Oncology, University Medical Centre Utrecht, Utrecht, The Netherlands; 2 Department of Pathology, University Medical Centre Utrecht, Utrecht, The Netherlands; 3 Department of Internal Medicine, University Medical Centre Utrecht, Utrecht, The Netherlands

## Abstract

**Background:**

Hypoxia inducible factor 1α (HIF-1α) plays an essential role in the adaptive response of cells to hypoxia and is associated with aggressive tumour behaviour. We have shown p27^kip1^, which is generally reduced in endometrial cancer, to be re-expressed in hypoxic regions. This possibly contributes to survival of cancer cells. The aim of this study was to evaluate the prognostic value of HIF-1α and p27^kip ^expression in patients with endometrioid endometrial cancer.

**Methods:**

Expression levels of HIF-1α, CAIX, Glut-1, and p27^kip1 ^were analyzed by immunohistochemistry. Percentage of positive cells, staining pattern (perinecrotic, diffuse, or mixed) and presence of necrosis were noted.

**Results:**

Necrosis was correlated with shortened disease free survival (DFS) (p *= *0.008) and overall survival (OS) (p *= *0.045). For DFS, perinecrotic HIF-1α expression was also prognostic (p *= *0.044). Moreover, high p27^kip1 ^expression was an additional prognostic factor for these patients with perinecrotic HIF-1α expression. In multivariate Cox regression, perinecrotic HIF-expression emerged as an independent prognostic factor. Perinecrotic HIF-1α expression was significantly associated with CAIX and Glut-1 expression, pointing towards functional HIF-1.

**Conclusions:**

In patients with endometrioid endometrial cancer, necrosis and necrosis-related expression of HIF-1α are important prognostic factors. More aggressive adjuvant treatment might be necessary to improve the outcome of patients with these characteristics.

## Background

Endometrial cancer is the most common malignant tumour of the female genital tract. The American Cancer Society estimated that 42160 women have been diagnosed with, and 7780 women have died of cancer of the female genital tract in 2009 in the US[1]. In the endometrium different subtypes of cancer can develop. Endometrioid endometrial carcinoma (EEC), or Type 1 cancers, are oestrogen dependent, often develop in a background of atypical complex hyperplasia and account for over 75% of cases. EEC patients generally have a good prognosis, with low mortality for stage 1 disease (5-year survival around 87%)[2]. However, survival drops in higher stage of disease. Additional prognostic factors could help to decide the need for adjuvant treatment and to identify new treatment strategies.

Solid tumours outgrow their own vasculature beyond the size of several mm^3^, resulting in hypoxia. Regions of necrosis are believed to demarcate regions of severe, chronic hypoxia[3]. Hypoxia is an important issue in carcinogenesis because it renders a more aggressive phenotype with increased invasiveness and proliferation, formation of metastases and poorer survival[4-8]. Besides, hypoxic malignant cells are more resistant to radiotherapy and chemotherapy[9-11]. In reaction to hypoxia, cells will alter their metabolism and activate certain survival genes. Hypoxia inducible factor 1 (HIF-1) plays an essential role in the adaptive cellular response to hypoxia[12,13]. HIF-1 is a transcription factor composed of the subunits HIF-1α and HIF-1β, which are basic helix-loop-helix DNA binding proteins. HIF-1β is constitutively expressed at the protein level. The activity of HIF-1 is predominantly regulated at the post-translational level by regulating HIF-1α protein stability. Under normoxia, HIF-1α is hydroxylated by prolyl hydroxylases in the oxygen dependent degradation domain. Hydroxylated HIF-1α is recognized by the Von Hippel Lindau protein, ubiquitinated, and destined for degradation by the proteasome. This process is inhibited during hypoxia[14], where stabilized HIF-1α heterodimerizes with HIF-1β to transactivate target genes after nuclear translocation by binding to the consensus Hypoxia Responsive Element (HRE) 5'-RCGTG-3' in promoters and enhancers[15]. Among these are growth factors, glucose transporters, glycolytic enzymes, and genes involved in gluconeogenesis, high-energy phosphate metabolism, erythropoiesis, haem metabolism, iron transport, vasomotor regulation and nitric oxide synthesis[13,15-17]. Protein products of these downstream genes help the cell to survive the hypoxic stress by increasing oxygen delivery (angiogenesis) and by switching to anaerobic glycolysis.

Previous breast cancer studies[6,7] showed that in hypoxic conditions, HIF-1α expression is seen perinecrotically, with induction of its target genes carbonic anhydrase 9 (CAIX), that plays a role in pH regulation[18] and Glut-1, a transmembrane glucose transporter [19,20]. In normoxic conditions, HIF-1α expression can also be induced by other mechanisms, but was to a much lesser extent associated with downstream activation. CAIX and Glut-1 thereby serve to identify functional HIF-1α[7]. In breast cancer, we showed that patients with a diffuse (non-hypoxia associated) HIF-1α staining pattern had a relatively better prognosis[7]. Elevated levels of CAIX are predictive of hypoxia in various types of cancer and are related to poor prognosis[21,22]. The prognostic value of CAIX and Glut-1 has not been studied in endometrial cancer. We previously suggested that HIF-1α plays an important role in endometrial carcinogenesis[23]. In postmenopausal woman, HIF-1α was increasingly overexpressed from inactive endometrium through hyperplasia to endometrioid carcinoma, paralleled by activation of its downstream genes. HIF-1α overexpression has further been shown to be correlated with a poorer survival in cancers of the brain[24], cervix[25] and ovary[26]. As HIF-1α is related to poor clinical outcome in some tumours, HIF-1α expression could be used to identify patients who are at risk of developing recurrent disease and who may benefit from adjuvant therapy. Moreover, targeting HIF-1α could be an attractive therapeutic strategy with the potential for disrupting multiple pathways crucial for tumour growth. In endometrial cancer, there are some conflicting data concerning the prognostic relevance of HIF-1α, probably due to varying methodology and patient groups[27-30]. Whether the different HIF-1α expression patterns have different prognostic implications in endometrial cancer is yet unknown.

In accordance with the need to decrease energy usage under low oxygen conditions, hypoxia induces cell-cycle arrest[31]. Cell-cycle arrest is regulated by complex interactions between cyclins, cyclin-dependent kinases (CDKs), and cyclin-dependent kinase inhibitors (CKIs)[32]. p27^kip1 ^is a CKI that regulates progression from G_1 _to S phase by inhibiting a variety of cyclin-CDK complexes. It has been shown that p27^kip1 ^expression is strongly reduced in endometrial cancer[33,34]. In a recent study on cell cycle regulation during endometrial carcinogenesis[23], p27^kip1 ^protein was re-expressed in necrotic (i.e. hypoxic) areas of endometrial carcinomas. We showed that p27^kip1 ^re-expression by hypoxia was HIF-1α-dependent and led to cell cycle arrest. This possibly contributes to survival of cancer cells in hypoxic parts of the tumour[35].

Several studies demonstrated that loss of the p27^kip1 ^protein, as assessed by immunohistochemistry, is a negative prognostic marker in malignancies[36,37]. However, the prognostic importance of HIF-1α induced re-expression of p27^kip1 ^is not known. Loss of p27^kip1 ^expression in endometrial carcinoma did not seem to correlate with worse prognosis in previous studies[38,39].

Understanding the mechanisms of carcinogenesis and progression of endometrial cancer is important as these insights might lead to improved diagnostic tools for the pathologist, improved prediction of prognosis and response to therapy, and eventually better biology-based disease management in the individual patient. The aim of this study was therefore to (1) re-evaluate the prognostic value of HIF-1α with emphasis on expression patterns and investigate the additional effect of p27^kip1 ^expression on predicting survival in EEC and (2) to determine relationships with the other clinicopathologic markers in the endometrioid type of endometrial cancer.

## Methods

### Patients and Tissues

The study group, diagnosed between 1992 and 2005 at the University Medical Centre Utrecht, The Netherlands, was composed as follows: a search in our tumour bank for patients with endometrioid endometrial cancer yielded 108 patients with paraffin embedded tumour tissue available. Patients with only endometrial sampling material available were not included in our study. Furthermore, patients were excluded if they had a second primary tumour of the cervix or ovary (n = 3), or a history of cervical carcinoma (n = 7). None of the patients received preoperative radio- or chemotherapy. Patient chart review was performed retrospectively; adequate follow-up data were lacking for 5 patients, leaving 93 patients. Table 1 gives an overview of the patient demographics and main pathological features. Haematoxylin and eosin-stained sections were revised and histologically graded. The tumour stage was defined by the International Federation of Gynaecologists and Obstetricians (FIGO) system. Anonymous use of redundant tissue for research purposes is part of the standard treatment agreement with patients in our hospital[40].

**Table 1:** Patient demographics and main pathological features of the group of endometrioid endometrial cancer patients (n = 93).

Variable	Grouping	N	(%)
Age	Mean	62.51	
	Minimum	31	
	Maximum	88	

Stage	I	54	(58)
	II	21	(23)
	III	13	(14)
	IV	5	(5)

Grade	1	28	(30)
	2	47	(51)
	3	18	(19)

Depth of myometrial invasion	0	3	(3)
	<50%	50	(54)
	>50%	40	(43)

Necrosis	Present	70	(75)
	Absent	23	(25)

HIF-1α expression	<1%	0	(0)
	≥1%	93	(100)
	<35%	74	(80)
	≥35%	19	(20)

HIF-1α expression pattern§	Purely Perinecrotic	20	(21)
	Purely Diffuse	39	(42)
	Mixed	34	(37)

CAIX	Positive	71	(76)
	Negative	22	(24)

Glut-1	Positive	76	(81)
	Negative	17	(19)

p27^kip1^	Positive	86	(93)
	Negative	7	(7)

Follow up time (for surviving patients, in months)	Minimum	13	
	Maximum	182	
	Mean	66	

Recurrence/Metastasis	Yes	18	(19)
	No	72	(78)
	Missing	3	(3)

Survival	Alive	71	(76)
	Death of endometrial cancer	11	(12)
	Death of intercurrent disease	10	(11)
	Cause of death unknown	1	(1)

§ Perinecrotic HIF present = mixed and purely perinecrotic HIF pattern grouped.

### Immunohistochemistry

Immunohistochemistry was performed on serial 4-μm tick paraffin slides. Table 2 presents all antibodies, dilutions, incubation times, and antigen-retrieval methods used. For all stainings, slides were deparaffinized with xylene and serial ethanol dilutions, and endogenous peroxidase activity was blocked in a buffer solution of pH 5.8 with hydrogen peroxide followed by antigen retrieval. For HIF-1α, antigen retrieval was performed with EDTA buffer pH 9.0 for 20 minutes at boiling temperature. After a cooling down period of 20 minutes, slides were incubated with a protein block (Vision Biosystems, Novocastra Laboratories Ltd, Newcastle Upon Tyne, United Kingdom) for 5 minutes, followed by anti-HIF-1α antibody (mouse monoclonal, BD Transduction Laboratories, dilution 1:50, overnight 4°C). Incubation with the secondary antibody (Post Primary block, Novocastra) for 30 minutes was preceded by incubation of Novolink™ polymer (Novocastra) for 30 minutes. For p27^kip1 ^staining, antigen retrieval was performed with citrate buffer, pH 6.0, for 20 minutes at boiling temperature. After a cooling down period of 20 minutes the slides were incubated with the primary antibody (Kip1/p27, BD Transduction Laboratories; dilution 1:500, overnight 4°C) followed by the secondary antibody (Powervision, ImmunoVision Technologies, Brisbane, CA, USA; ready to use, 30 minutes) as before[35]. For CAIX and Glut-1 staining protocols were used as before[6,7,23]. Slides were developed with diaminobenzidine for 10 minutes, followed by haematoxylin counterstaining. In between steps, slides were washed in PBS. Before the slides were mounted, all sections were dehydrated in alcohol and xylene. Appropriate positive and negative controls were used throughout.

**Table 2:** Overview of the antibodies used and tissue processing details.

Primary Anti Body	Type Anti Body	Source	Dilution	Antigen Retrieval	Second step	Positive control	Incubation time/temp (primary antibody)	Procedure
HIF-1α	MoAb	Transduction	1:50	EDTA, Ph9.0, 20 minutes, 93°C	NV	mamma	o/n 4°C	By hand

Glut-1	PoAb, Rabbit	DAKO	1:200	Citrate, Ph6.0, 20 minutes, 93°C	G-AR IgG + strep 1	Placenta	60 minutes/room temp	Autostainer

CAIX	PoAb, Rabbit	Abcam	1:1000	Citrate, Ph6.0, 20 minutes, 93°C	PV	Grawitz tumour	60 minutes/room temp	By hand

p27^kip1^	MoAb	Transduction	1:500	Citrate, Ph6.0, 20 minutes, 93°C	PV	Skin	o/n 4°C	By hand

HIF-1α = hypoxia-inducible factor-1α; Glut-1 = glucose transporter-1; CAIX = carbonic anhydrase IX; MoAb = monoclonal antibody; PoAb = polyclonal antibody; Transduction = BD Transduction Laboratories, BD Biosciences, San Diego, CA, USA; DAKO = DAKOCytomation, Glostrup, Denmark; Abcam = Abcam, Cambridge Science Par, Cambridge, UK; G-aR IgG = biotinylated Goat-anti Rabbit IgG (BA-1000, Vector laboratories, CA, diluted 1:500) + strep 1 = Streptavidin peroxidase labelling (Streptavidin HRP IM0309, Beckman Coulter, diluted 1:100); NV = Novolink™ polymer (Novocastra); PV = Powervision ready to use (Poly-HRP-anti Ms/Rb/RtlgG biotin free, ImmunoLogic, ImmunoVision technologies, Brisbane CA, USA).

### Evaluation of Staining

Scoring was performed by two observers (PJvD, LS) as before[6,7,23]. Stainings were scored blinded to clinicopathologic data and results of other stainings. For HIF-1α and p27^kip1^, the percentage of dark, homogenously stained nuclei was estimated, ignoring cytoplasmic staining. Both were considered positive when ≥1% staining was seen. Glut-1 and CAIX were considered positive when membrane staining was seen. The pattern of HIF-1α, Glut-1 and CAIX was noted as purely diffuse (throughout the tumour without emphasis on areas with necrosis, thought to be due to non-hypoxic stimuli), perinecrotic (only positive staining around a necrotic area, thought to be hypoxia induced) or a combination of these two ("mixed"). No double staining was performed as this was done previously in breast cancer, and no further topographic analysis of staining was performed[7]. For p27^kip1^, the pattern of staining was noted as perinecrotic, centrally in tumour islands (as these may be pre-necrotic areas), diffuse or mixed.

### Statistical Analysis

The Chi-square test or Fisher's Exact test (when appropriate) were used to evaluate correlations between HIF-1α expression, presence of necrosis, p27^kip1 ^expression and stage, grade, myometrial invasion. Correlations between HIF-1α, CAIX and Glut-1 expression were evaluated with McNemar's test and Spearman rank correlation coefficient. Mann-Whitney-U was used to assess HIF-1α expression and age at time of diagnosis. For univariate survival analysis, the HIF-1α staining pattern or the 75% percentile (35%) was chosen as cut-off value for HIF-1α and p27^kip1 ^expression, closely corresponding to the proportion of patients that remained alive and well (81%). Kaplan-Meier curves were plotted, and differences between the curves were analyzed with the log-rank test to test disease-free survival (DFS) and overall survival (OS). Cox proportional hazard was used for the multivariate survival analysis entering stage, grade, myometrial invasion, age, perinecrotic HIF-1α and p27^kip1 ^expression as covariates. Two sided uncorrected p-values < 0.05 were considered statistically significant. All analysis were performed with SPSS for Windows version 15.0; SPSS Inc., Chicago, IL, USA.

## Results

### HIF-1α and necrosis

**Figure 1:**
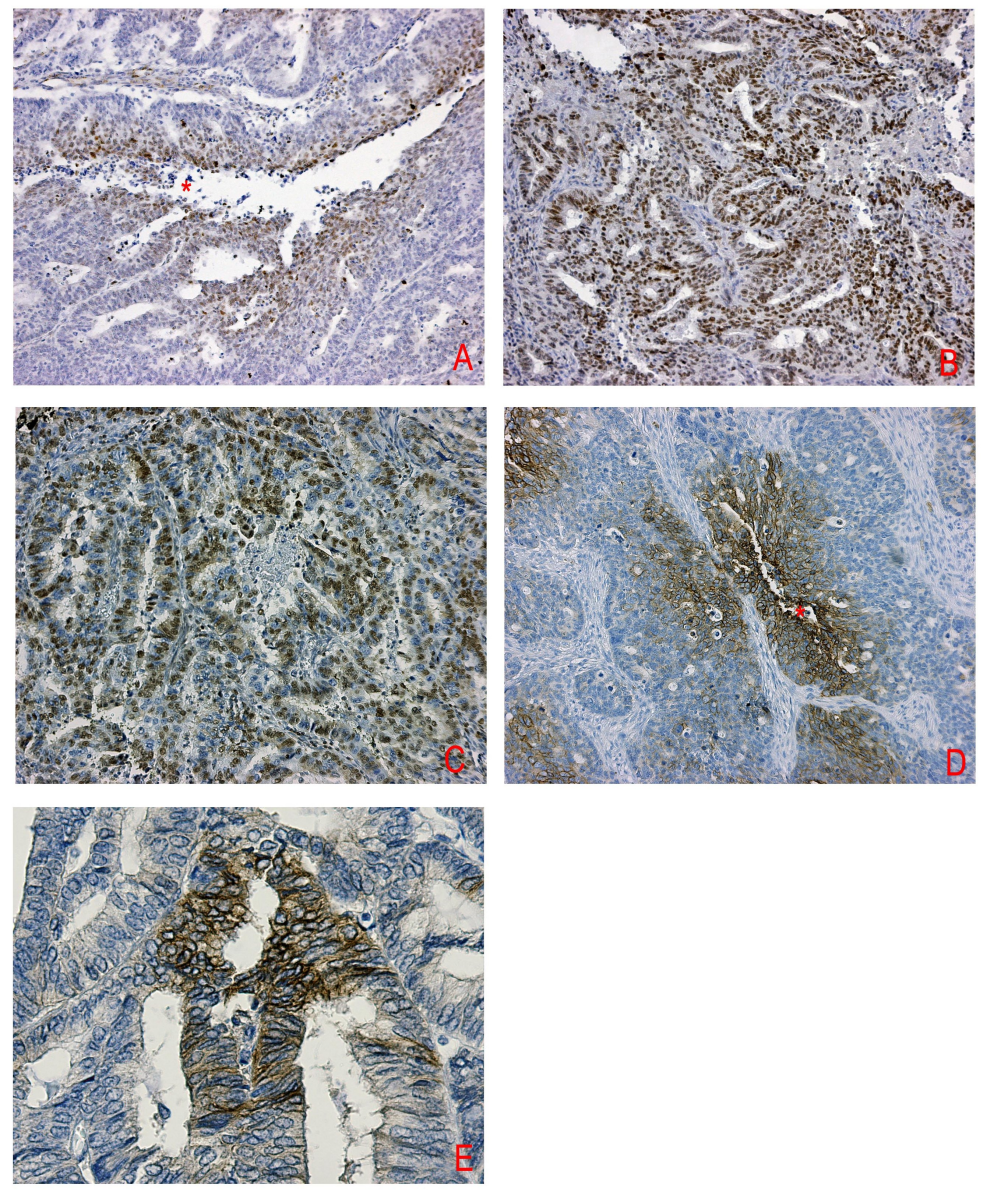
**Immunohistochemical staining of HIF-1α, CAIX, Glut-1 and p27^kip1 ^in endometrioid endometrial carcinoma**. Typical patterns are shown: A) perinecrotic HIF-1α expression (10× magnification). B) Diffuse HIF-1α expression (10× magnification). C) Diffuse p27^kip1 ^expression (10× magnification). D) Perinecrotic Glut-1 expression (10× magnification) E) Membrane CAIX expression (20× magnification). Asterisk indicates necrosis.

Detectable levels of HIF-1α were found in all endometrial carcinomas. The mean level of nuclear staining for HIF-1α was 26% (median, 20%; range, 1-90%). 20/93 cases (21%) showed a purely perinecrotic staining pattern, 39/93 (42%) a purely diffuse pattern, and 34/93 (37%) showed a mixed (diffuse and perinecrotic) pattern (figure 1). Necrosis was found in 73/93 (75%) of the endometrial carcinomas. Not all tumours with necrosis showed perinecrotic HIF-1α expression.

Presence of perinecrotic HIF-1α expression was significantly associated with high-grade tumours (table 3). Nine out of 28 tumours (32%) showed perinecrotic HIF-1α expression in grade 1 tumours compared with 70% grade 2 and grade 3 tumours (χ^2^-test, p = 0.004). HIF-1a expression in endometrioid endometrial cancer was not stage dependent and there was no association of perinecrotic HIF-1α expression and depth of myometrial invasion or age. Similarly, necrosis was more often seen in high grade than in low grade tumours (χ^2^-test, p = 0.003). No association was found between necrosis and tumour stage, depth of myometrial invasion or age.

**Table 3:** Associations between HIF-1α staining pattern, clinicopathological features and expression of CAIX, Glut-1 and p27^kip1^.

Perinecrotic HIF-1α				
		**Yes (%)**	**No (%)**	**P-value**

Stage	I	30 (56)	24 (44)	
	II	12 (57)	9 (43)	0.845
	III	9 (69)	4 (31)	
	IV	3 (60)	2 (40)	

Grade	1	9 (32)	19 (68)	
	2	33 (70)	14 (30)	0.004
	3	12 (67)	6 (33)	

Myometrial Invasion	0	0	3 (100)	
	<50%	29 (58)	21 (42)	0.107
	>50%	25 (63)	15 (37)	

Necrosis	Yes	54 (77)	16 (23)	<0.0001
	No	0	23 (100)	

Recurrence/Metastasis	Yes	14 (79)	4 (22)	0.043
	No	37 (51)	35 (49)	

p27^kip1^	Pos	48 (89)	38 (97)	0.125
	Neg	6 (11)	1 (3)	

p27^kip1^	≤50%	48 (58)	35 (42)	0.896
	>50%	6 (60)	4 (40)	

p27^kip1 ^expression pattern	Peri/central	30(91)	3(9)	<0.0
	Other*	24(40)	36(60)	001

CAIX	Pos	47 (67)	24 (33)	0.004
	Neg	7 (32)	15 (68)	

Glut-1	Pos	48 (63)	28 (37)	0.035
	Neg	6 (35)	11 (65)	

CAIX and Glut-1	Pos	42 (68)	20 (32)	0.007
	Neg	12 (39)	19 (61)	

HIF-1α = hypoxia-inducible factor 1α; CAIX = carbonic anhydrase IX; Glut-1 = glucose transporter 1, * other staining patterns include diffuse staining or negative for p27^kip1^.

### p27^kip1 ^protein expression

Expression of p27^kip1 ^was found in 86/93 (93%) of endometrial carcinomas (figure 1). The mean level of nuclear staining for p27^kip1 ^was 24% (median, 20%; range 0-100%). There was no correlation between percentage p27^kip1 ^positive cells and stage, grade, age, HIF-1α expression pattern or necrosis (additional file 1). In 56% of the tumours (30/54) with perinecrotic HIF-1α expression, we observed central/perinecrotic p27^kip1 ^staining (McNemar; p < 0.0001). p27^kip1 ^staining pattern did not correlate with age, stage or myometrial invasion. However, perinecrotic/central p27^kip1 ^expression pattern was correlated with higher grade (p = 0.046).

### CAIX and Glut-1 expression in relation with HIF-1α

CAIX was expressed in 71/93 (76%) cases. Glut-1 showed membranous expression in 76/93 (81%) cases. Positive expression of both CAIX and Glut-1 was seen in 68% of the perinecrotic HIF-1α expressing tumours compared to 32% of the diffuse group (χ^2^-test, 0.007). Perinecrotic HIF-1α expression was also significantly associated with CAIX or Glut-1 expression (McNemar; p = 0.004 respectively p < 0.0001).The 54 cases with any perinecrotic HIF-1α expression showed positive expression of CAIX or Glut-1 in 47/54 and 48/53 cases, respectively. Diffuse HIF-1α expression was accompanied by CAIX expression in 24/39 or Glut-1 expression in 28/39 cases. Levels of HIF-1α expression correlated with expression levels of CAIX ( Spearman; p = 0.012) but not with expression levels of Glut-1.

### Survival analysis results

**Figure 2:**
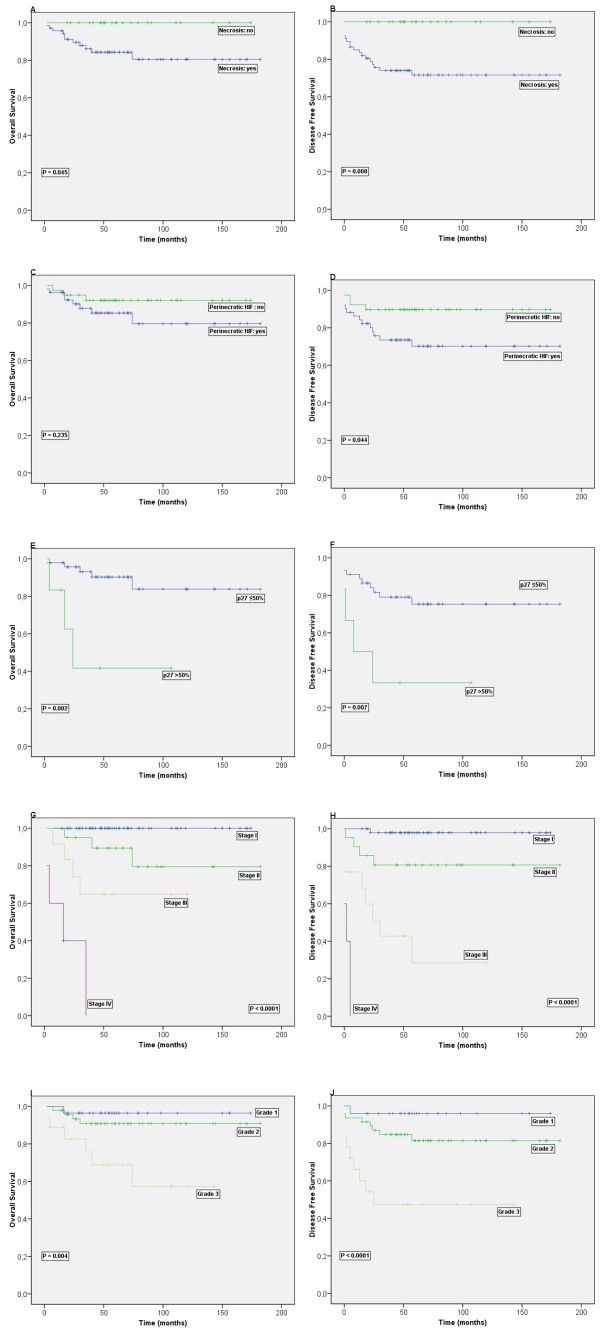
**Kaplan-Meier survival curves illustrating disease-free survival and overall survival for endometrioid endometrial carcinoma patients (n = 93) with tumour necrosis (A, B) and patients with perinecrotic type HIF-1α expression (C, D). **E and F show the survival for the patients with perinecrotic HIF-1α expression (n = 51) and high p27^kip1 ^expression. Stage and grade show prognostic value as expected in G - J.

Locoregional recurrences (vaginal, pelvic, or both) or distant metastases were found in 18 of 93 patients. Three patients with unknown recurrence status were censored for disease free survival analysis. Stage and grade showed prognostic value as expected, underlining the representativeness of the patient group (see Figure 2). Univariate analysis (Kaplan-Meier, log rank test) for the whole group of patients showed that necrosis was correlated with shortened disease free survival (DFS) (p *= *0.008) and overall survival (OS) (p *= *0.045). For DFS, perinecrotic HIF-1α expression also was prognostic (p *= *0.044), as shown in figure 2. HIF expression in more than 35% of cells was associated with a shorter OS (p = 0.032). CAIX expression was not correlated with survival. Membranous Glut-1 expression however correlated with a shorter DFS (p = 0.037). Subgroup analysis of low stage (stage I and II) (n = 75) patients did show perinecrotic HIF-1α expression also to be an indicator of shorter disease free survival in this patient group (p = 0.033), while significance was lost in the stage III/IV (n = 18) subgroups (p = 0.737).

P27^kip1 ^expression or expression pattern had no significant relation to prognosis. Subgroup analysis of patients with perinecrotic HIF-1α expression (*n *= 55) revealed that high p27^kip1 ^expression (>50% positive cells) was an additional prognostic factor indicating a poor prognosis for DFS (p = 0.007) and OS (p = 0.002) in this subgroup.

In a multivariate model together with stage, grade, depth of myometrial invasion and age at diagnosis, only perinecrotic HIF-1α expression emerged as an additional prognostic factor to stage and age for DFS (p = 0.044; RR: 0.312; 95% Confidence Interval 0.100-0.972). Multivariate analysis of patients with perinecrotic HIF-1α expression, showed high p27^kip1 ^to be a prognostic factor for DFS above stage and age (p = 0.011; RR: 6.735 95% Confidence Interval 1.557-29.122).

## Discussion

Hypoxia and its key regulator HIF-1α have been shown to play an important role in endometrial carcinogenesis[23], but contradictory results have been published as to the prognostic value of HIF-1α overexpression in endometrial carcinoma[27-30], while expression patterns have been ignored. The aim of this study was therefore to re-evaluate the prognostic value of HIF-1α overexpression in a representative group of patients with endometrioid endometrial cancer, with emphasis on expression patterns. Also, as p27^kip1 ^is re-expressed in hypoxic regions of EEC[35], we investigated the additional effect of p27^kip1 ^expression on predicting survival in EEC. HIF-1α overexpression, especially the perinecrotic type, and necrosis appeared to be independent indicators of poor prognosis. High p27^kip1 ^(>50% positive cells) expression was an additional prognostic factor in the subgroup of patients with perinecrotic type of HIF-1α expression.

Our results on the prognostic value of HIF-1α overexpression are in line with those of Sivridis et al.[27] where HIF-1α was associated with a shorter overall survival in stage 1 endometrial cancer. However, others did not find a significant prognostic impact of HIF-1α overexpression[28-30]. Immunohistochemical HIF-1α studies are difficult to compare because of a variation in the definition of HIF-1α positivity. Previous studies did not consider the different expression patterns throughout the tumours (diffuse versus perinecrotic) that have been shown in other cancers to be prognostically crucial[7]. Another difference between the present and previous studies is the cut off value for HIF-1α expression. In the present study, the cut off value for prognostic value of HIF-1α was 35%, much higher than in other studies where the cut off varied between 1% and 5%[6,41]. Our results indicate that also in endometrial cancer, the pattern of HIF-1α expression is more important for the prognosis than percentage and intensity of HIF-1α expressing cells in general. This significance of expression pattern could be explained by the fact that perinecrotic HIF-1α expression is thought to be hypoxia driven, whereas diffuse HIF-1α expression may rather be due to non-hypoxic stimuli[6,7]. In a previous study from our group[23] we showed that diffuse HIF-1α expression was associated with the highest microvessel density (MVD); perinecrotic and mixed patterns were associated with an intermediate MVD (p < 0.05). This indicates that diffuse HIF-1α expression is more likely to be due to non-hypoxic stimuli than to non-necrosis associated hypoxia. We showed that perinecrotic HIF-1α expression is more often accompanied by activation of its downstream factors Glut-1 and CAIX, indicating it to be more active than diffuse HIF-1α. More activation of HIF-1α and its target genes would give more tumour cells a survival advantage in a hypoxic environment. Perinecrotic HIF-1α expression retained its prognostic significance in the subgroup of low stage, while significance was lost in stage III/IV patients, indicating that HIF-1α expression seems to be especially important in low stage patients. However, sample size of the subgroups (especially the high stages) was limiting. Therefore, no definitive conclusions on the prognostic value of HIF-1α in high stage patients can be made. Loss of p27^kip1 ^expression has been reported for a number of human tumour types and has been correlated with poor prognosis and tumour aggressiveness[36,42]. Only one study showed a correlation between p27^kip1 ^expression and prognosis in endometrial cancer[43]. However, we found no prognostic influence of global p27^kip1 ^expression in endometrioid endometrial carcinoma. This is in line with the outcome of others[38,39,44]. Since we found hypoxia to be able to induce re-expression of p27^kip1 ^in a HIF-1α dependent way in a previous study[35], we evaluated the prognostic value of p27^kip1 ^in relation to HIF-1α in the present study. In the subgroup of patients with perinecrotic HIF-1α expression, high p27^kip1 ^expression (>50% positive cells) was associated with a shorter DFS and OS. This fits with our previously proposed model that hypoxia induced re-expression of p27^kip1 ^may result in dormancy of hypoxic cells, which in combination with HIF-1α induced expression of genes regulating supply of energy, growth factors and other survival factors, may promote cellular survival and adaptation of sub clones within the tumour that may contribute to metastatic disease and poor clinical outcome. In our previous study however, only central/perinecrotic p27^kip1 ^expression was associated with HIF-1α induced re-expression[35]. In the present study, central/perinecrotic p27^kip1 ^expression was still associated with perinecrotic HIF-1α expression; however this subgroup was too small to correlate with survival data.

Interestingly, all patients lacking tumour necrosis survived, and none of these patients developed a locoregional or distant recurrence. The prognostic value of necrosis is in concordance with previous results by Scholten et al.[45] who showed that necrosis is a significant prognostic factor in Stage I-III endometrioid endometrial carcinoma. Although necrosis is a strong independent prognostic factor for endometrial carcinoma, its clinical use may be limited because of a moderate reproducibility[45-47]. Further, excluding the presence of necrosis requires adequate sampling. More objective biomarkers like perinecrotic HIF-1α expression could result in a higher reproducibility.

## Conclusions

We conclude that in endometrioid endometrial carcinoma, absence of necrosis is a favourable prognostic factor. Tumours with a perinecrotic HIF-1 expression are more aggressive than tumours with purely diffuse HIF-1 expression. Furthermore, the combination of perinecrotic HIF expression and high p27^kip1 ^expression is indicative for an unfavourable prognosis. More aggressive adjuvant treatment might be necessary to improve the outcome of these patients. To this end, targeting the HIF pathway might provide an attractive strategy to treat hypoxic and highly angiogenic tumours. Thus far, selective HIF-1 inhibitors have not been identified. A number of non-selective inhibitors, which indirectly target signaling pathways upstream or downstream of HIF-1 are known to decrease the key regulating HIF-1α protein levels. Preliminary results show encouraging results for these single agent treatments, however severe toxicities have been encountered. Lack of specificity increases the difficulty in attributing any anti-tumorigenic effects of these drugs specifically to inhibition of HIF-1. Further studies are needed to show whether adaptation of therapy protocols might be of benefit for endometrial cancer patients with perinecrotic HIF-1α overexpression.

## Competing interests

The authors declare that they have no competing interests.

## Authors' contributions

LS and NH contributed equally to the manuscript. LS collected samples, performed immunohistochemistry, analysed data, carried out data interpretation and drafted the manuscript. NH participated in the conception and design of the study, collected samples, performed immunohistochemistry, and drafted the manuscript. PG provided technical support and performed immunohistochemistry. EW and RV participated in design of the study and critically revised the manuscript. PD participated in the conception and design of the study, performed revision and new histological staging of samples, supervised statistics and critically revised the manuscript. All authors read and approved the final manuscript.

## Pre-publication history

The pre-publication history for this paper can be accessed here:

http://www.biomedcentral.com/1471-2407/10/307/prepub

## Supplementary Material

Additional file 1 **Associations between p27**^**kip1 **^**staining pattern, clinicopathological features, necrosis and HIF-1α expression pattern**. Click here for file
