# Supplementary material for: Necrosis related HIF-1α expression predicts prognosis in patients with endometrioid endometrial carcinoma
Source: BMC Cancer. 2010 Jun 19;10:307. doi: 10.1186/1471-2407-10-307 (PMC2909981; doi:10.1186/1471-2407-10-307)
Supplement: Additional file 1 — Associations between p27kip1 staining pattern, clinicopathological features, necrosis and HIF-1α expression pattern. [file 1471-2407-10-307-S1.DOC]

## Additional file 1

| **P27^kip1^ expression** | | | | |
| --- | --- | --- | --- | --- |
|  | | **Positive (%)** | **Negative (%)** | **P-value** |
| Stage | I  II  III  IV | 50 (93)  20 (95)  11 (85)  5 (100) | 4 (7)  1 (5)  2 (15) 0 | 0.617 |
| Grade | 1  2  3 | 26 (93)  43 (92)  17 (94) | 2 (7)  4 (9)  1 (6) | 0.918 |
| Myometrial Invasion | 0  <50%  >50% | 3 (100)  47 (94)  36 (90) | 0  3 (6)  4 (10) | 0.683 |
| Age | ≤ 62  >62 | 42 (91)  44 (94) | 4 (9)  3 (6) | 0.488 |
| Necrosis | Yes  No | 63 (90)  23(100) | 7 (10)  0 | 0.15 |
| HIF-1α expression | Perinecrotic  Diffuse | 48 (89)  38 (97) | 6 (11)  1 (3) | 0.125 |

## Associations between p27^kip1^ staining pattern, clinicopathological features, necrosis and HIF-1α expression pattern.
